# Notes from Practice

**Published:** 1877-12

**Authors:** 


### NOTES FROM PRACTICE.

---

*Abscess of Antrum.*—W. P. called to have his teeth examined. Upon examination it was found that the first superior left molar was abscessed, and discharged into the antrum, and that the outer plate of the superior maxilla had absorbed, letting pus through the cheek. The opening in the bone was about half an inch below, and a little to the left of the infraorbital foramen. Upon inquiry I ascertained from the patient that it had been discharging for a year or more. With the consent of the patient the tooth was extracted, the antrum treated through the opening made by the extraction of the tooth—and the opening on the cheek healed up very prettily in three or four weeks.

Shortly before this opening appeared on the cheek, the young man received a blow on the same cheek with a base ball. After the opening appeared he consulted his physician, who introduced a probe into the antrum through the opening on the cheek and pronounced it a case of necrosis of the superior maxlilary and sent the young man to a surgeon in Philadelphia, to have it operated on. The surgeon declined to interfere, saying that it would get well without an operation.

I think they both made a mistake in their diagnosis, and furthermore that the blow received from the ball had little or nothing to do with the abscess.—J. O. H.

---

*A Question of Articulation.*—What is the best method of articulating a double set of teeth, so as to be of the most service and comfort to the wearer:

Say whether the buccal surfaces of the molars should come even, or should that surface of the superior teeth overlap the same surface of the inferior teeth. Have had some trouble recently where the buccal surfaces were even. Any hint that will be of service to the patient will be thankfully received.

ANSWER.—Double cases are not difficult to articulate correctly if care is taken that the plates on which the bite is taken are well fitted and are of such material as will not change its shape. Wax plates are bad on this account; and gutta-percha, though ordinarily used, is not sufficiently inflexible. The modeling composition of S. S. White's is the best substance for this purpose. It is easily moulded, and is not softened by the heat of the mouth; with it the ridges are easily built up to the required shape and length, and a thin layer of soft wax on the surface serves to unite the two plates after their accurate adjustment Difficulty is often experienced iu double sets from the fact that the width of the lower jaw at the posterior part of the plate is greater than that of the upper, *i. e.* from one heel of the plate to the other. It is better here to leave the lower teeth in rather than the upper ones out.

If difficulty is experienced on account of the cheek being caught and bitten between the last molars, it is well to leave these off; this will also prevent the disagreeable rattling of the teeth together which is so annoying to the wearer and his or her associates. It is well to be cautious of carrying the plate back too far; the opposite error is more safe.

---

*Removing Salivary Calculus from Sub-lingual Gland*—A gentleman about 40 years of age. applied to me,—stating he had been suffering with a painful swelling under the tongue for several weeks, and therefore was unable to eat solid food. He said he had "applied to several physicians for treatment, without getting any relief, and he wished me to examine his mouth, aud tell him what I thought was the trouble." Upon examination of the swelling, I found to the right of the frænum of the tongue, the swelling more prominent, and sensitive to touch. I took a keen sharp pointed bistoury and cut down to the calculus and felt the grating of it under the point of the instrument I then told him what it was, and that it was the cause of the trouble.

He seemed surprised and incredulous, that a stone should form there. I prevailed on him to let me cut an opening large enough to let it out, which I did, and found the concretion the size of a navy bean of a yellowish white color; he expressed himself the most grateful individual I ever saw, and said he was free from pain immediately on its removal. This is the second case I have had, and only ones I ever saw. The first case was a much older, and corpulent subject, and the concretion removed from that case, was the size of a hazel nut.

